# Exenterative Surgery in Advanced Pelvic Cancer: Survival Outcomes and an Exploratory Predictive Modeling Framework for Surgical Decision Support

**DOI:** 10.3390/diagnostics16162595

**Published:** 2026-08-16

**Authors:** Elena Chitoran, Vlad Rotaru, Aisa Gelal, Daniela-Cristina Stefan, Giuseppe Gullo, Traian Patrascu, Laurentiu Simion

**Affiliations:** 1 Medicine School, “Carol Davila” University of Medicine and Pharmacy, 050474 Bucharest, Romania; 2 General Surgery and Surgical Oncology Department I, Bucharest Institute of Oncology, “Prof. Dr. Alexandru Trestioreanu”, 022328 Bucharest, Romania; 3 Department of Obstetrics and Gynecology, Villa Sofia Cervello Hospital, University of Palermo, 90146 Palermo, Italy; 4 General Surgery Department, “Dr. I. Cantacuzino” Clinical Hospital, 030167 Bucharest, Romania

**Keywords:** pelvic exenteration, advanced pelvic cancer, oncologic outcomes, overall survival, predictive modeling, surgical decision support, R0 resection, patient selection, clinical prediction model, risk stratification, decision-making in oncology

## Abstract

**Background/Objectives:** Pelvic exenteration is among the most aggressive procedures in surgical oncology and remains associated with substantial morbidity and highly variable oncologic outcomes. Achieving negative resection margins (R0) is the strongest determinant of long-term survival, yet accurate preoperative prediction of resectability remains difficult. This study evaluated oncologic outcomes following pelvic exenteration and explored the feasibility of a predictive modeling framework for estimating the probability of achieving R0 resection using routinely available pretherapeutic variables. **Methods:** A retrospective single-center cohort study included 229 patients undergoing pelvic exenteration for advanced pelvic disease between 2008 and 2024. Survival outcomes were analyzed using Kaplan–Meier and Cox regression methods. An exploratory machine-learning framework based on Random Forest (RF) and Extreme Gradient Boosting (XGB) algorithms was developed using demographic, oncologic, radiologic, therapeutic, and biological preoperative variables to estimate the likelihood of achieving negative surgical margins. **Results:** Median overall survival (OS) was 40 months (95%CI: 37–42 months), while median progression-free survival (PFS) was 18 months (95%CI: 17–19 months). Recurrent disease was associated with significantly worse oncologic outcomes, whereas colorectal malignancies demonstrated superior survival compared with urologic and gynecologic tumors. Multivariable analysis identified resection-margin status as the only independent predictor significantly associated with both OS (HR 1.338, 95%CI 1.211–1.764, *p* = 0.0137) and PFS (HR 3.499, 95%CI 2.357–5.195, *p* < 0.0001). RF achieved higher apparent overall accuracy (83.6%) but failed to identify positive-margin cases. XGB demonstrated more balanced discriminatory performance, with an ROC-AUC of 0.792 and improved identification of incomplete resections. Absence of radiologically visible loco-regional lymphadenopathy emerged as the strongest predictor of achieving R0 resection. **Conclusions:** Pelvic exenteration continues to provide meaningful oncologic benefit in selected patients when R0 resection is achievable. Although the predictive models remain exploratory and are not suitable for direct clinical implementation, they demonstrate the feasibility of integrating multidimensional pretherapeutic variables into decision-support frameworks for patient selection.

## 1. Introduction

Pelvic exenteration remains one of the most radical procedures in oncologic surgery and is generally reserved for patients with advanced or recurrent pelvic disease in whom complete resection may still provide a meaningful survival benefit. Since its initial description, the procedure has evolved from a predominantly palliative intervention into a potentially curative strategy for highly selected patients with locally advanced pelvic malignancies. Improvements in perioperative care, reconstructive techniques, imaging quality, and multimodal oncologic treatment have contributed to reduced perioperative mortality and improved long-term outcomes in experienced centers [1]. More broadly, advances in oncologic surgical techniques and perioperative management have progressively expanded the range and complexity of resections that can be safely considered in selected patients [2,3].

Despite these advances, pelvic exenteration continues to be associated with considerable physiologic burden, substantial postoperative morbidity, prolonged recovery, and major implications for quality of life [4,5,6]. The decision to proceed with exenterative surgery therefore requires careful balancing of oncologic benefit against procedural risk. This challenge is particularly relevant given the heterogeneity of pelvic malignancies, variability in disease extent, prior treatment exposure, and differences in institutional expertise.

Depending on the anatomical extent and the pelvic organs involved, pelvic exenteration is conventionally classified as anterior, posterior, or total, with the specific organs included in each procedure varying according to patient sex and tumor origin.

Achieving negative surgical margins (R0 resection) has consistently emerged as the strongest predictor of long-term survival following pelvic exenteration [1,7]. Multiple studies have demonstrated that margin status is closely associated with recurrence risk and overall survival, reinforcing the importance of accurate patient selection and preoperative assessment of resectability. However, predicting the feasibility of complete oncologic resection remains difficult because resectability is influenced by a complex interaction of tumor biology, anatomical extension, treatment response, and patient-specific factors [8,9,10].

Although survival outcomes after pelvic exenteration have been described in several institutional and collaborative series, there remains limited integration of predictive analytics into preoperative decision-making. Existing prognostic models are often constrained by disease-specific cohorts, small sample sizes, or narrow variable selection, limiting their ability to reflect the multidimensional reality of multidisciplinary oncologic assessment [11,12,13]. Furthermore, few studies have explored how routinely available pretherapeutic data could be used to estimate the probability of achieving complete resection before surgery.

Recent advances in predictive modeling and machine-learning methodologies have generated growing interest in their application within surgical oncology [14,15,16]. Rather than replacing clinical judgment, such approaches may provide complementary decision-support tools capable of identifying patterns not easily captured through conventional statistical methods alone. In procedures characterized by high morbidity and profound functional consequences, predictive frameworks may contribute to more individualized estimation of benefit, particularly when integrated into multidisciplinary treatment planning.

The present study aimed to evaluate long-term oncologic outcomes following pelvic exenteration in a large single-center cohort and to investigate the feasibility of constructing an exploratory predictive framework capable of estimating the probability of achieving R0 resection using routinely available pretherapeutic variables. By combining survival analysis with predictive modeling, this study proposes a clinically grounded approach toward improving patient selection in highly invasive pelvic oncologic surgery.

## 2. Materials and Methods

### 2.1. Study Design

This study was designed as a retrospective single-center cohort analysis of patients undergoing pelvic exenteration for advanced pelvic disease at the Bucharest Oncology Institute “Prof. Dr. Alexandru Trestioreanu” between January 2008 and June 2024. All adult patients with a history of pelvic malignancy who underwent pelvic exenteration during the study period were considered eligible for inclusion. The study adopted a consecutive inclusion strategy in order to minimize selection bias and to reflect real-world surgical practice. Patients were included regardless of surgical intent, including both curative and palliative procedures, and irrespective of whether the disease was primary, recurrent, or synchronous. All patients underwent some form of pelvic exenteration, a major procedure performed under general anesthesia by experienced oncological surgeons and other specialists, as appropriate (urologists, vascular surgeons). All efforts were made to ensure R0 resection, including resection of adjacent diseased organs, pelvic plane resection, or regional lymphadenectomy as required.

All eligible patients were included in the descriptive analysis, whereas a predefined subset was used for survival analysis and predictive modeling (patients with synchronous tumors or patients previously treated for pelvic cancers with complex fistulas between pelvic organs but no current signs of recurrence) were excluded due to increased risk of bias. Follow-up continued until death, loss to follow-up, or censoring of results on 30 June 2025, thus allowing for a minimum follow-up interval of 12 months for included patients.

Ethical approval was obtained from the institutional review board and the study was conducted in accordance with the Declaration of Helsinki.

### 2.2. Patient Population and Data Collection

Data were obtained from a prospectively maintained institutional surgical registry supplemented by retrospective review of electronic and paper medical records.

Clinical, demographic, oncologic, surgical, pathological, and follow-up information was extracted from patient charts, operative reports, pathology records, imaging investigations, and outpatient follow-up documentation.

Baseline variables included demographic characteristics, oncologic diagnosis, histopathologic findings, disease extent, treatment history, and survival outcomes. Tumors were categorized according to origin as urologic, gynecologic, colorectal, or other pelvic malignancies. Disease status was classified as primary or recurrent. Histopathologic variables included tumor differentiation grade, lymphovascular invasion, and perineural invasion where available either on preoperative biopsy or resected specimen. Preoperative imaging findings were reviewed to assess loco-regional extension, lymph node involvement, pelvic floor invasion, vascular or neural infiltration, extra-pelvic spread, and presence of ascites.

Patients were discussed within a multidisciplinary tumor board before treatment allocation, and neoadjuvant therapy was administered according to disease-specific protocols and institutional practice.

According to the anatomical compartments involved, pelvic exenteration may be classified as anterior, posterior, or total. In women, anterior pelvic exenteration involves resection of the urinary and genital organs, whereas posterior pelvic exenteration involves resection of the genital organs together with the rectum. In men, anterior pelvic exenteration corresponds to radical cystoprostatectomy, involving removal of both the urinary bladder and prostate. Posterior pelvic exenteration is not conventionally defined as a distinct exenterative procedure in men, as the corresponding resection would essentially consist of rectal resection or abdominoperineal rectal amputation, which is classified as a colorectal procedure rather than a pelvic exenteration. Total pelvic exenteration involves complete multivisceral resection of the pelvic organs in both sexes.

Margin status was recorded according to pathological examination, with R0 resection defined as microscopically negative margins, while R1 and R2 resections indicated microscopic and macroscopic residual disease, respectively.

Procedural characteristics were recorded primarily to contextualize surgical burden and treatment heterogeneity and do not represent a focus of this study. Postoperative variables, including complications and adjuvant therapies, were intentionally excluded from predictive modeling because the objective of the framework was to simulate real-world pretherapeutic decision-making using only information available prior to surgical intervention.

### 2.3. Outcome Definitions

The primary outcome of the study was overall survival. Secondary oncologic outcomes included progression-free survival, recurrence patterns, and the probability of achieving complete oncologic resection (R0).

Overall survival was defined as the interval between pelvic exenteration and death from any cause or last available follow-up. Progression-free survival was defined as the interval between surgery and radiologic, clinical, or histopathologic evidence of recurrence.

Because the principal objective of the study was to explore patient selection and oncologic benefit, perioperative technical variables and postoperative morbidity were considered descriptive contextual measures rather than primary analytical outcomes.

Perioperative morbidity was not included as a primary analytical outcome; however, postoperative complications, reinterventions, length of hospital and intensive care unit stay, and 30-day mortality were descriptively reported to provide appropriate clinical context regarding the surgical burden of pelvic exenteration.

### 2.4. Statistical Analysis

Continuous variables were tested for normality using the Shapiro–Wilk and Kolmogorov–Smirnov tests. Normally distributed variables were expressed as mean ± standard deviation, whereas non-parametric variables were summarized using medians and interquartile ranges.

Comparisons between groups were performed using Student’s *t*-test or Mann–Whitney U testing for continuous variables and chi-square or Fisher’s exact tests for categorical variables. Correlation analyses were performed using Spearman’s rho and Kendall’s tau coefficients where appropriate.

Survival distributions were estimated using Kaplan–Meier methodology, with comparisons performed using the log-rank test. Univariable Cox proportional hazards regression was used to assess the association between individual clinical, pathological, and surgical variables and OS or PFS. Results are reported as hazard ratios (HRs) with 95% confidence intervals (Cis) and *p*-values. Variables included in multivariable analysis were selected based on biological plausibility, literature relevance, and significance in univariate testing. Statistical significance was defined using a two-sided *p*-value < 0.05.

Survival analyses were restricted to patients with active malignant disease. Patients undergoing pelvic exenteration for benign conditions, as well as those presenting with synchronous malignancies, were excluded from OS and PFS analyses to avoid introducing etiologically heterogeneous or non-oncologic survival patterns.

### 2.5. Development of the Predictive Modeling Framework

In parallel with survival analysis, an exploratory predictive modeling framework was developed to evaluate whether routinely available pretherapeutic information could estimate the probability of achieving negative resection margins (R0). The target variable to be predicted, designated as Marg_rez, was a binary variable encoding negative resection margins as 0 and carcinomatous infiltration of the resection margins as 1.

This predictive endpoint was selected because negative surgical margins represent the strongest modifiable determinant of long-term survival after pelvic exenteration and therefore constitute a clinically meaningful surrogate of oncologic benefit.

Rather than relying on preselection based solely on univariate significance, all available pretherapeutic variables considered clinically meaningful were incorporated into exploratory modeling in order to reflect real-world multidisciplinary decision-making. This inclusive strategy was chosen not only to incorporate established prognostic factors but also to allow identification of potentially underrecognized predictors that may emerge from complex interactions among clinical, radiologic, and biologic features.

Predictive variables were grouped into six domains: demographic, oncologic, histopathologic, radiologic, therapeutic, and biological. Demographic predictors included age, sex, and residence area. Oncologic variables included tumor origin, primary versus recurrent disease, TNM stage, metastatic status, and loco-regional disease extent. Histologic variables included tumor differentiation grade, lymphovascular invasion, perineural invasion, and inflammatory infiltrate. Radiologic predictors included nodal disease, pelvic floor invasion, lateral extension, neurovascular involvement, ascites, and extra-pelvic spread. Therapeutic variables included neoadjuvant treatment, treatment response, and urgency of surgery. Biological variables included routinely available laboratory markers reflecting inflammatory, nutritional, hepatic, renal, and coagulation status.

To improve model consistency and reduce bias, structured preprocessing of the dataset was applied prior to machine-learning analysis. Cases involving benign pathology and synchronous malignancies were excluded because of their potential to confound oncologic interpretation and margin assessment. These benign cases were retained exclusively for descriptive characterization of the complete surgical cohort and were excluded from the oncologic survival analyses and predictive modeling. Given the retrospective nature of the dataset, missing values and heterogeneous coding required standardization. Categorical variables were converted into binary representations, while exenteration subtype was encoded using one-hot encoding. Missing continuous variables were imputed using median substitution, whereas categorical variables were imputed using the most frequent category. After preprocessing, the final predictive cohort consisted of 220 patients.

Two supervised machine-learning algorithms were selected for exploratory predictive analysis: Random Forest and Extreme Gradient Boosting (XGB). Random Forest was chosen because of its ability to model nonlinear relationships and tolerate heterogeneous clinical variables, while XGB was selected because of its strong classification performance in structured datasets [17].

The dataset was divided into 75% training data and 25% testing data, with stratification performed according to the target variable in order to preserve class proportions.

Random Forest models were initialized using 500 decision trees, with parameter optimization performed through internal cross-validation. XGB hyperparameters, including learning rate, maximum depth, subsampling ratio, and feature sampling fraction, were optimized using a constrained grid-search strategy adapted to the relatively limited sample size. Internal validation was performed using repeated cross-validation procedures k-fold (k = 5) for optimal hyperparameter selection and prevention of overfitting [18].

Model performance was evaluated using area under the receiver operating characteristic curve, sensitivity, specificity, classification accuracy, and feature importance analysis. In addition to evaluating the overall performance of the optimized predictive model, the relative importance of individual variables was also extracted. Feature importance was estimated using the model-specific importance metrics implemented by each algorithm: mean decrease in impurity (Gini importance) for Random Forest and gain-based feature importance for XGBoost. These values allowed derivation of a hierarchy of major factors influencing the probability of achieving radical resection.

Model calibration was additionally assessed on the held-out test set using calibration curves and the Brier score, with lower Brier scores indicating better overall probabilistic accuracy. Given the limited number of R1/R2 events in the test set, calibration curves were constructed using five quantile-based probability groups to reduce instability associated with sparsely populated bins.

### 2.6. Conceptual Integration into Therapeutic Decision-Making

The predictive framework was intentionally developed as an exploratory proof-of-concept rather than a clinically deployable algorithm. The purpose was to investigate whether preoperative clinical, biologic, and imaging-derived features could estimate the likelihood of achieving negative surgical margins before operative intervention.

Given the highly invasive nature of pelvic exenteration and its substantial impact on postoperative quality of life [19,20], patient selection remains critical. Within this context, predictive modeling may contribute to multidisciplinary decision-making by identifying patients with a higher probability of meaningful oncologic benefit through R0 resection while potentially avoiding futile or excessively morbid procedures in cases with limited resectability.

Although the present model should be regarded as an early exploratory framework rather than a mature clinical algorithm, it illustrates a feasible methodological approach for integrating predictive analytics into pelvic exenteration decision-making. Predictive analytics were conceptualized as an adjunct to multidisciplinary oncologic evaluation rather than a replacement for clinical or surgical judgment.

The broader implementation of predictive decision-support tools in pelvic exenteration will likely depend on validation within large multicenter datasets, such as those provided by the PelvEx Collaborative [21,22,23,24,25], which may enable development of more generalizable and clinically reliable models.

### 2.7. Methodological Considerations

The findings of this study should be interpreted within the context of several limitations. The retrospective monocentric design introduces inherent selection bias and limits generalizability. The cohort includes heterogeneous malignancies accumulated over a prolonged period during which surgical strategies and oncologic treatments evolved. Temporal evolution of imaging modalities, perioperative management, and systemic oncologic therapies throughout the study period may also have influenced observed outcomes.

The predictive framework was developed using a relatively limited dataset, increasing the risk of overfitting and model instability. No external validation cohort was available. Additionally, the relatively imbalanced distribution between R0 and R1/R2 cases may have influenced classifier behavior and contributed to reduced sensitivity for identifying incomplete resections. Consequently, the predictive analysis should be regarded as exploratory and hypothesis-generating rather than immediately applicable to clinical practice. The framework is intended to provide a methodological basis for future validation using larger multicenter collaborative datasets. Within this framework, R0 feasibility was considered a clinically relevant decision-support target because of its consistent association with long-term oncologic survival.

The inclusion of malignancies of different pelvic origins introduces biological and therapeutic heterogeneity that may influence the predictive signal captured by the models. Although tumor origin was incorporated as a preoperative predictor and an additional subgroup analysis was performed to explore its association with resection status, the limited sample size and, particularly, the small number of R1/R2 cases within individual tumor categories precluded the development of adequately powered tumor-specific machine-learning models. Such models should be investigated in substantially larger multicenter datasets.

## 3. Results

### 3.1. Baseline Characteristics of Study Cohort

A total of 229 patients were considered eligible for inclusion in the study. The mean age at the time of pelvic exenteration was 60 years (standard deviation ± 11.5, ranging from 24 to 87 years). The age histogram shows a nearly normal distribution, relatively centered around the range of 55–70 years, consistent with the typical ages of onset of pelvic cancers. Male to female ratio was 1/1.89.

Exenterative surgery was indicated primarily as the therapeutic option for advanced pelvic malignant tumors (224 cases—97.82%), but also for 5 cases (2.18%) in which the patient was previously treated for a pelvic tumor, does not have current clinical or imaging signs of recurrence and suffers from a complex fistula between multiple pelvic organs.

All resected surgical specimens were analyzed, and histopathological reports were reviewed during data collection. Among the cancer patients 133 (58.08%) had a primary tumor, 87 (37.99%) had pelvic recurrence of disease after initial treatment, and 4 (1.75%) had synchronous tumors with distinct histology.

**Figure 1:**
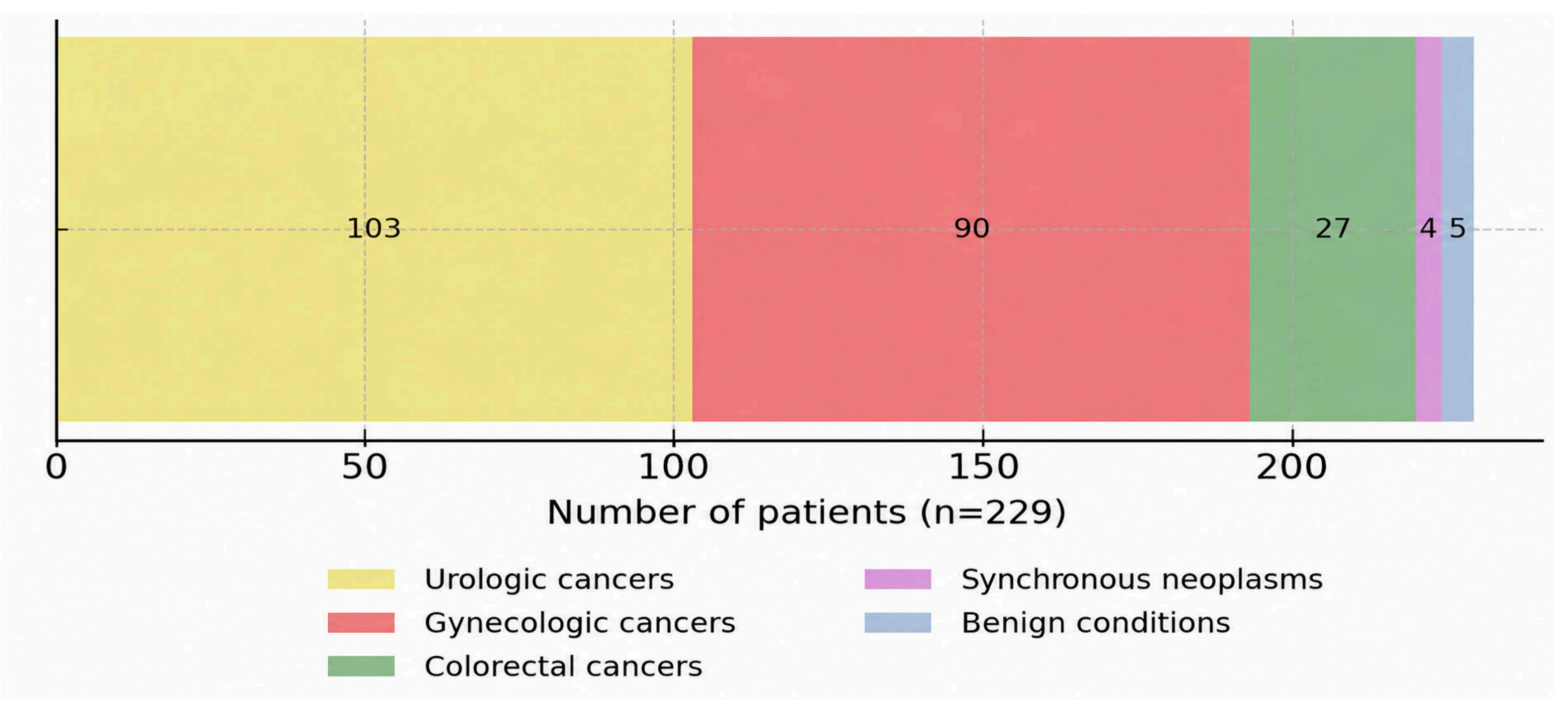
Structure of the cohort according to the indication for pelvectomy.

The origin of the neoplastic disease was most often a locally advanced or recurrent urological cancer (44.98%—103 patients). Pelvic exenteration was indicated in 90 cases of gynecological cancers (39.30%) and 27 cases of colorectal cancers (11.79%). The etiology of the gynecological tumors was cervical cancer in 56 cases, endometrial cancer in 17 cases, ovarian cancer in 15 cases and vaginal cancer in 2 cases. A detailed structure of the cohort according to the indication for pelvectomy is presented in Figure 1.

The most frequent histological types were urothelial carcinoma (37.12%) and squamous cell carcinoma (30.57%), followed by adenocarcinoma (18.34%). Six cases had very aggressive sarcoma-type histologies (3.02%). Tumors were poorly differentiated in over half of the cases (55.46%).

Regarding the type of exenterative procedure performed, anterior pelvic exenteration was required in 148 cases (64.63%), consistent with the predominantly urologic origin of the treated malignancies. Total pelvic exenteration was performed in 48 patients (20.96%), while the remaining 33 patients (14.41%) underwent posterior exenteration.

In 15 cases (6.55%), surgery was performed on an emergency basis because of acute hemorrhagic, septic, or obstructive complications. Fifty patients (21.83%) presented with complex pelvic fistulas preoperatively. Resection of the pelvic floor was necessary in 18 cases (7.86%), whereas in 211 cases (92.14%) resection was performed in a supralevator plane. Lateral extension of resection, consisting of segmental resection of the external iliac artery and/or vein with vascular reconstruction, was required in four cases. No patient required resection of major neural structures.

Additional extra-pelvic organ resection was necessary in 20.09% of cases and involved between one and four organs, including segmental ileal resection (20 cases), appendectomy (9 cases), colectomy (4 cases), vulvectomy (2 cases), omentectomy/peritoneal resection (4 cases), muscular resections (4 cases), and nephrectomy (1 case). Regional lymphadenectomy was performed in 173 patients (75.55%), including bilateral pelvic, para-aortic, bilateral inguinal dissections, or combinations thereof. Regional lymphadenectomy was not considered necessary in 56 patients.

Restoration of gastrointestinal continuity was achieved in 81 cases either through colorectal anastomosis, preferred whenever technically feasible, or terminal colostomy when anastomosis was not possible because of inadequate vascularization, poor tissue quality secondary to prior radiotherapy, or patient-related factors such as severe obesity or protein malnutrition. In 13 cases, the colorectal anastomosis was hand-sewn, while in 10 cases it was mechanically stapled.

Urinary reconstruction was performed in 196 cases and consisted predominantly of bilateral cutaneous ureterostomy in a “double-barrel” configuration (129 cases, 65.82%). Other urinary diversion techniques included ileal conduit urinary diversion according to the Bricker technique (46 cases, 23.47%) and ileal neobladder reconstruction (21 cases, 10.71%). The high proportion of cutaneous ureterostomies observed in our cohort should be interpreted in the context of both the specific characteristics of the study population and our institutional surgical practice. Urologic malignancies represented the predominant tumor group (56.33%), with anterior pelvic exenteration consequently being the most frequently performed procedure (64.63%). Cutaneous ureterostomy was also preferentially used as a simpler urinary diversion in selected elderly or frail patients, particularly when minimizing additional reconstructive complexity and operative time was considered desirable. The choice of urinary diversion was individualized according to patient characteristics, surgical complexity, and the availability of specialized urologic reconstructive expertise. These factors collectively account for the relatively high rate of cutaneous ureterostomy observed in our series.

All included patients were evaluated in a multidisciplinary tumor board and appropriate neoadjuvant therapies were proposed and administered to patients based on the particularities of the case. As a result, 48.03% of patients (110) received neoadjuvant therapy consisting of chemotherapy, radiotherapy, brachytherapy or a combination thereof. Following surgery, all cases were reassessed within the multidisciplinary tumor board, and adjuvant therapy (chemotherapy, radiotherapy, immunotherapy, or a combination thereof) was considered necessary in 176 patients.

Postoperative morbidity was observed in 118 patients (51.53%), with some patients experiencing multiple complications at different time points. Immediate complications (postoperative days 0–7) occurred in 68 patients (29.69%) and included predominantly pulmonary, hepatorenal, thromboembolic, hemorrhagic, septic, digestive, and wound-related events. Early complications (postoperative days 8–30) occurred in 54 patients (23.58%), most commonly including bowel obstruction, hemorrhagic or septic complications, digestive fistula or perforation, and urinary diversion-related events. Late complications (>30 days) were recorded in 70 patients (30.57%) and predominantly consisted of incisional hernia, bowel obstruction, septic complications, digestive or genitourinary fistulas, and urinary diversion dysfunction. Surgical reintervention was required in 57 patients (24.89%), including 32 (13.97%) during the immediate or early postoperative period and 25 (10.92%) during later follow-up. Thirty-day postoperative mortality was 2.18% (5 patients), resulting predominantly from septic complications with subsequent multiple-organ failure or acute cardiovascular events. The median hospital stay was 18 days, while the median intensive care unit stay was 5 days.

### 3.2. Radicality of Resection

Based on the preoperative imaging findings available at the time of surgical planning, the procedure was performed with curative intent in 190 cases (82.89%). In the remaining 39 cases, surgery was classified preoperatively as palliative due either to the presence of distant metastatic disease or invasion of surgically unresectable structures, including osseous, vascular, or neural involvement. Nevertheless, all efforts were made to achieve radical resection whenever technically feasible. Even in metastatic cases, optimal pelvic cytoreduction without residual pelvic disease was pursued whenever possible, which was achieved in 18 of the 39 palliative cases. This strategy aimed not only to prevent subsequent local progression-related complications but also to create the possibility of future curative treatment of secondary lesions in selected oligometastatic patients.

**Figure 2:**
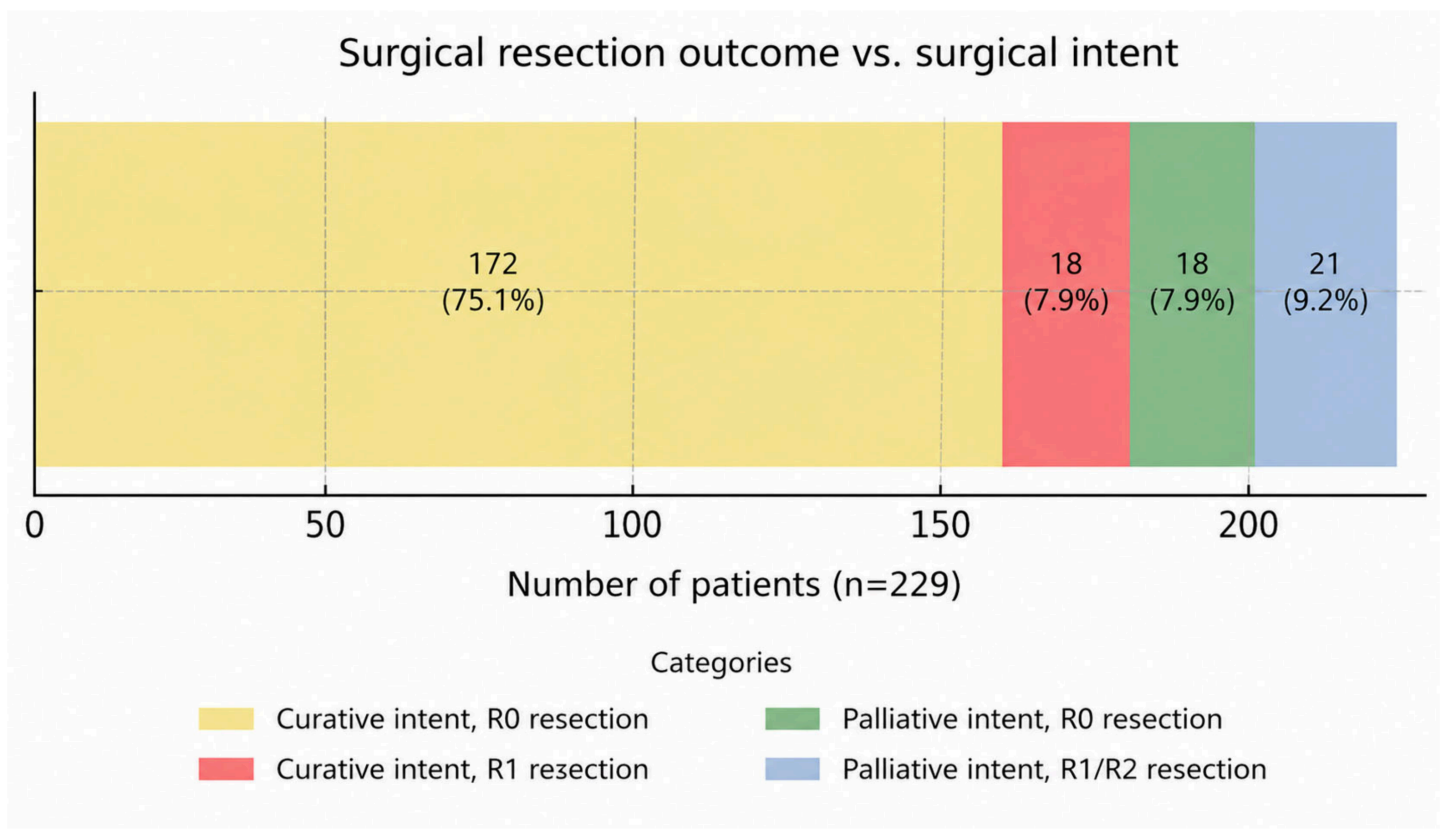
Radicality—surgical resection outcome vs. surgical intent.

Another important consideration is that, despite curative surgical intent, negative resection margins could not be achieved in 18 of the 190 procedures initially classified as radical, as histopathological examination demonstrated microscopic or macroscopic residual disease (R1/R2 resection). Consequently, a truly radical resection was ultimately achieved in 172 cases (75.11%) and was more likely to be feasible in the absence of extensive lateral disease extension, including vascular or osseous invasion and extensive parametrial or pararectal involvement (Figure 2).

### 3.3. Survival Analysis

The median postoperative follow-up period was 31 months (range: 12–120 months). Follow-up was concluded either at the time of patient death or when the patient was no longer available for follow-up evaluation. Overall survival data were obtained using the CNAS (Romanian National Health Insurance House) insured-patient verification platform, which allows verification of patient vital status (alive/deceased). For patients listed as deceased who no longer attended follow-up visits, family members were contacted by telephone in order to establish the exact date of death. Continuation of the telephone interview after explanation of its purpose was considered as consent for data collection. Patients for whom the exact date of death could not be obtained were censored at the time of loss to follow-up or at the end of the study follow-up period (June 2025).

Progression-free survival (PFS) analysis was based on dates of disease progression confirmed clinically, radiologically, or through histopathologic verification. Similar to overall survival (OS), patients were censored at the time of loss to follow-up or at the completion of the study follow-up period.

Kaplan–Meier analysis demonstrated a median overall survival (OS) of 40 months (95% CI: 37–42 months) and a median progression-free survival (PFS) of 18 months (95% CI: 17–19 months). Within our cohort, four patients may be considered effectively cured from an oncologic perspective, remaining alive at 120 months following surgery without evidence of local recurrence or systemic disease.

A subgroup analysis was performed for patients undergoing total pelvic exenteration. Among the 45 eligible patients, R0 resection was achieved in 28 cases (62.2%), compared with 154 of 175 patients (88.0%) undergoing other types of pelvic exenteration (Fisher’s exact *p* = 0.00021). Median OS was 42 months (95% CI 36–45) following total pelvic exenteration and 44 months (95% CI 39–46) following other exenterative procedures, with no significant difference between groups (log-rank *p* = 0.283). Median PFS was 21 months (95% CI 14–28) versus 24 months (95% CI 22–26), respectively, with a modest but statistically significant difference (log-rank *p* = 0.039).

**Figure 3:**
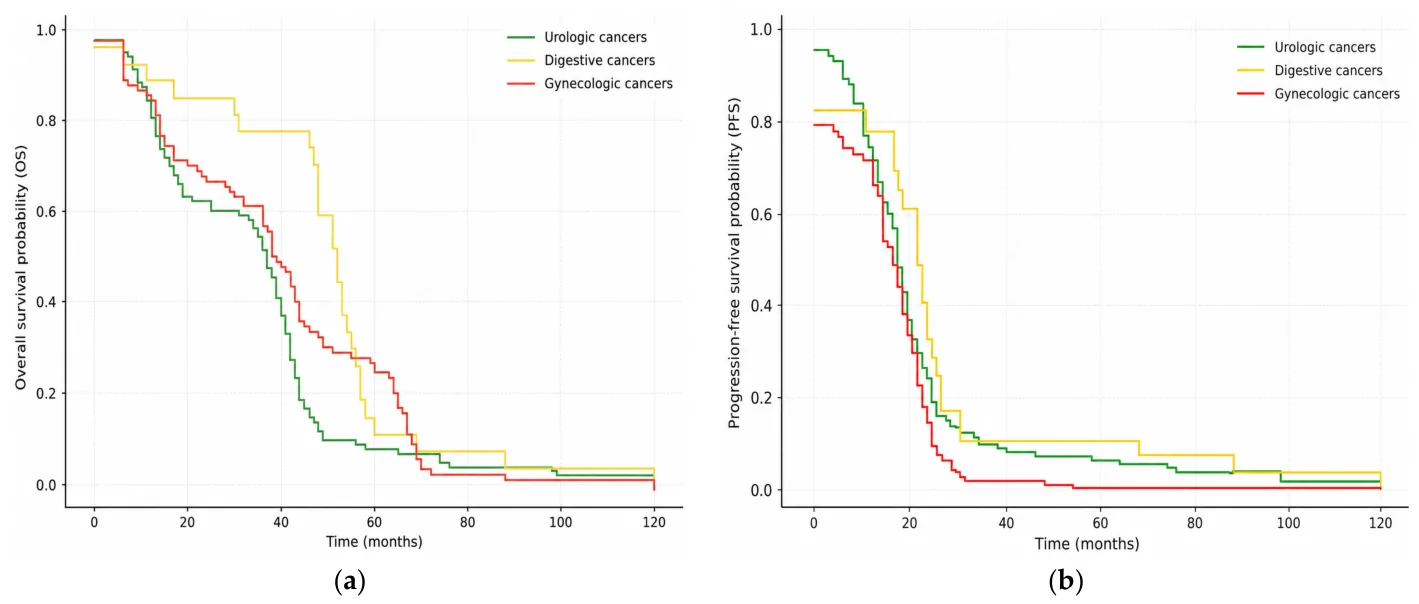
Kaplan–Meier curves for overall survival (OS) and progression-free survival (PFS) according to the origin of the treated tumor: (**a**) OS; (**b**) PFS.

Stratified survival analysis according to tumor origin revealed significant differences between oncologic subgroups. Patients with colorectal malignancies demonstrated a median overall survival (OS) of 52 months (95% CI: 47–55 months), significantly superior (log-rank *p* = 0.0098) to patients with urologic cancers (37 months) and gynecologic cancers (38 months). Progression-free survival (PFS) followed a similar pattern (22 vs. 18 vs. 17 months, respectively; log-rank *p* = 0.00378). These findings suggest a more favorable oncologic prognosis in colorectal malignancies, potentially related to improved resectability and differential response to neoadjuvant and adjuvant therapies (Figure 3).

**Figure 4:**
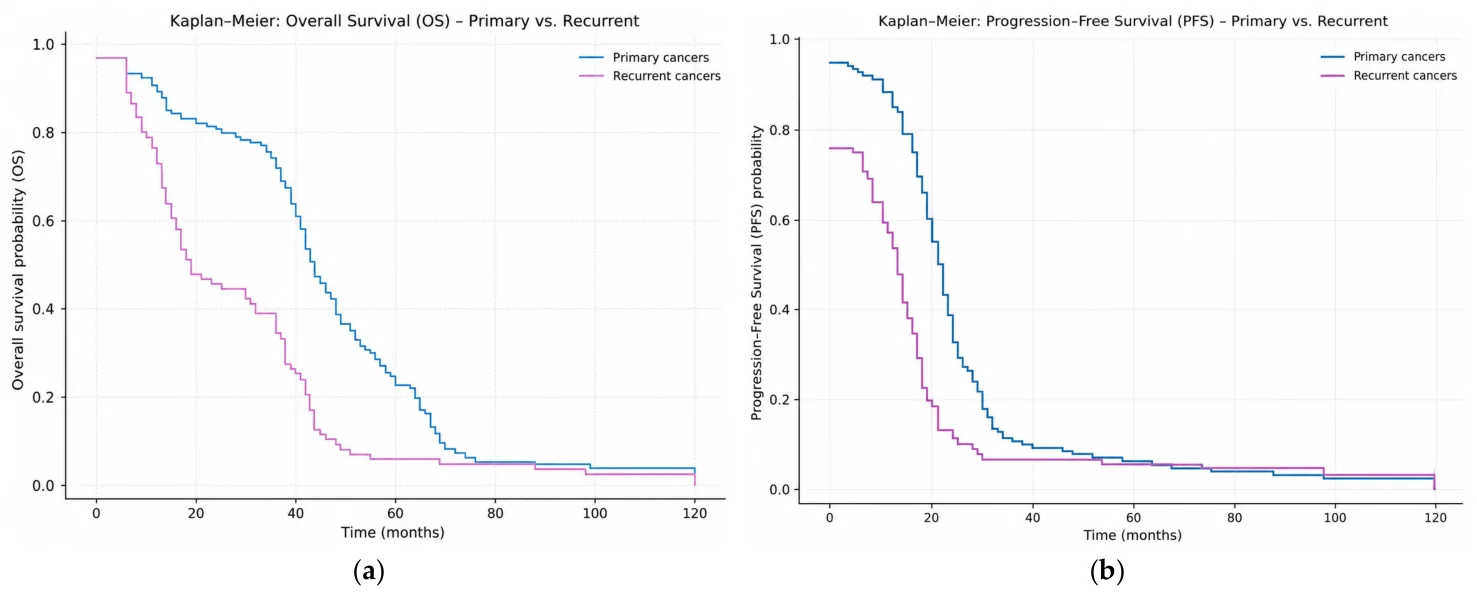
Kaplan–Meier curves for overall survival (OS) and progression-free survival (PFS) according to the recurrent vs. primary status of the treated tumor: (**a**) OS; (**b**) PFS.

The most pronounced differences were observed between primary tumors and recurrent disease. Median overall survival (OS) for patients with primary tumors was 44 months (95% CI: 41–48 months), compared with only 19 months (95% CI: 15–32 months) in patients with recurrent tumors (*p* < 0.0001). Progression-free survival (PFS) followed a similar trend (21 vs. 13 months, respectively; *p* < 0.0001)—Figure 4. These findings confirm the major negative prognostic impact of pelvic tumor recurrence, where distorted anatomy, the effects of prior treatments, and increased biological aggressiveness reduce both the likelihood of achieving radical resection and the effectiveness of adjuvant therapies.

Univariable Cox regression identified several factors associated with OS and/or PFS, as summarized in Table 1. Positive resection margins were significantly associated with both OS and PFS, while extra-pelvic organ resection, TNM stage, vascular involvement, LVI, PNI, and peritumoral lymphocytic infiltrate were significantly associated with PFS.

However, after multivariable analysis, resection-margin status remained the only independent predictor significantly associated with both overall survival and progression-free survival (OS: HR 1.338, 95% CI 1.211–1.764, *p* = 0.0137; PFS: HR 3.499, 95% CI 2.357–5.195, *p* < 0.0001).

### 3.4. Exploratory Predictive Modeling Analysis

**Figure 5:**
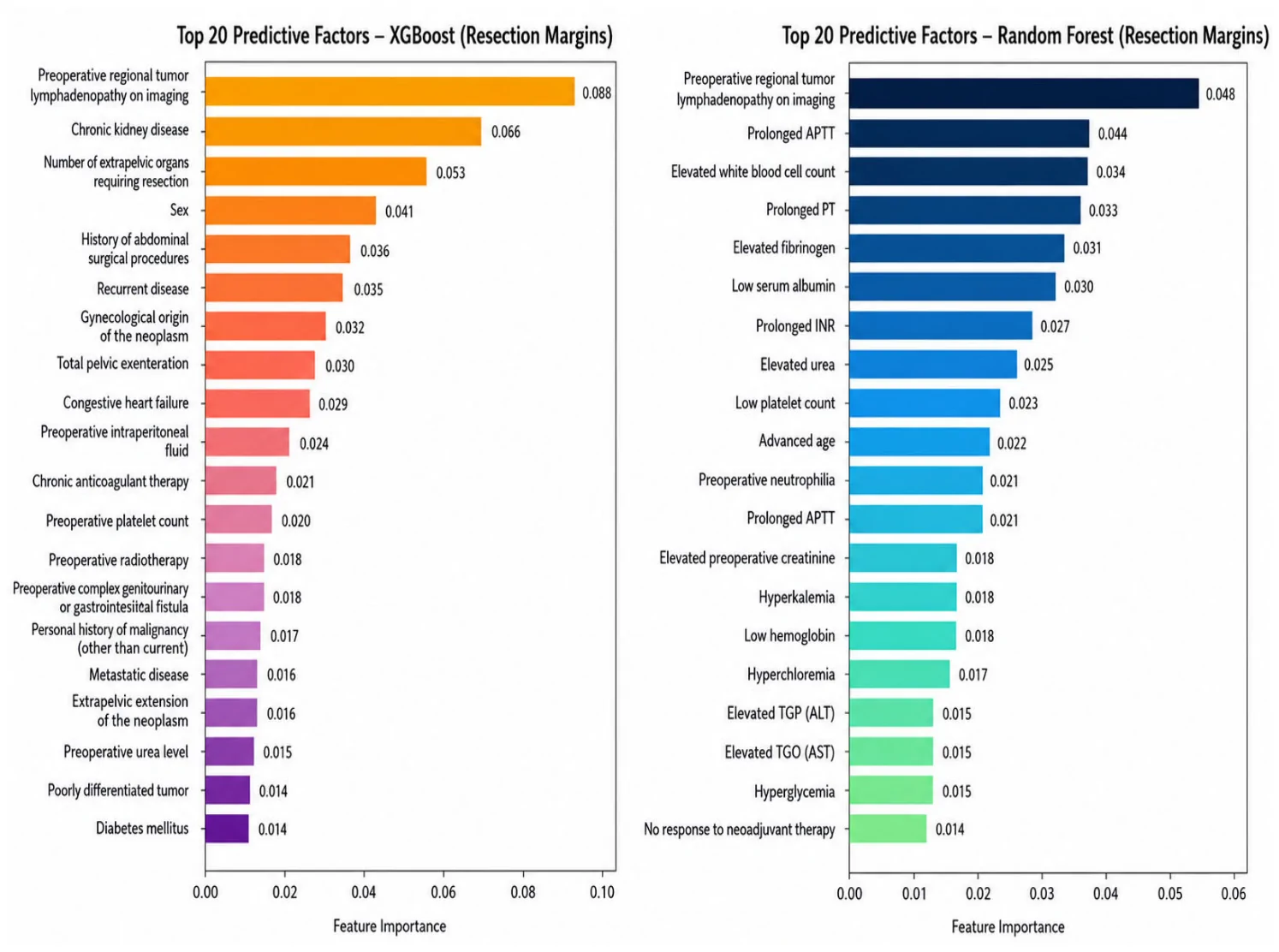
The importance of the top 20 predictive variables for XGB and RF models.

Application of the two machine-learning models, Random Forest (RF) and Extreme Gradient Boosting (XGB), to the analyzed cohort demonstrated notable differences in their ability to predict radical resection (R0). Table 2 presents the comparative performance metrics of the two predictive models. Figure 5 presents the top 20 predictive factors identified as important by each model together with their relative contribution values.

Sensitivity (recall) represents the proportion of correctly identified positive cases among all actual positive cases and is calculated as: true positive/(true positive + false negative). Specificity represents the proportion of correctly identified negative cases among all actual negative cases and is calculated as: true negative/(true negative + false positive). Precision (positive predictive value) represents the proportion of correctly predicted positive cases among all predicted positive cases and is calculated as: true positive/(true positive + false positive). Accuracy represents the proportion of all correct predictions among all evaluated cases and is calculated as: (true positive + true negative)/(true positive + true negative + false positive + false negative). Balanced accuracy represents the arithmetic mean of sensitivity and specificity and is used to compensate for the limitations of conventional accuracy in imbalanced datasets where one class predominates. F1-score represents the harmonic mean of precision and recall and is particularly useful in class-imbalanced datasets. ROC-AUC represents the area under the receiver operating characteristic curve and reflects the model’s ability to discriminate between the two classes across varying decision thresholds, ranging from 0.5 (random prediction) to 1.0 (perfect discrimination).

**Figure 6:**
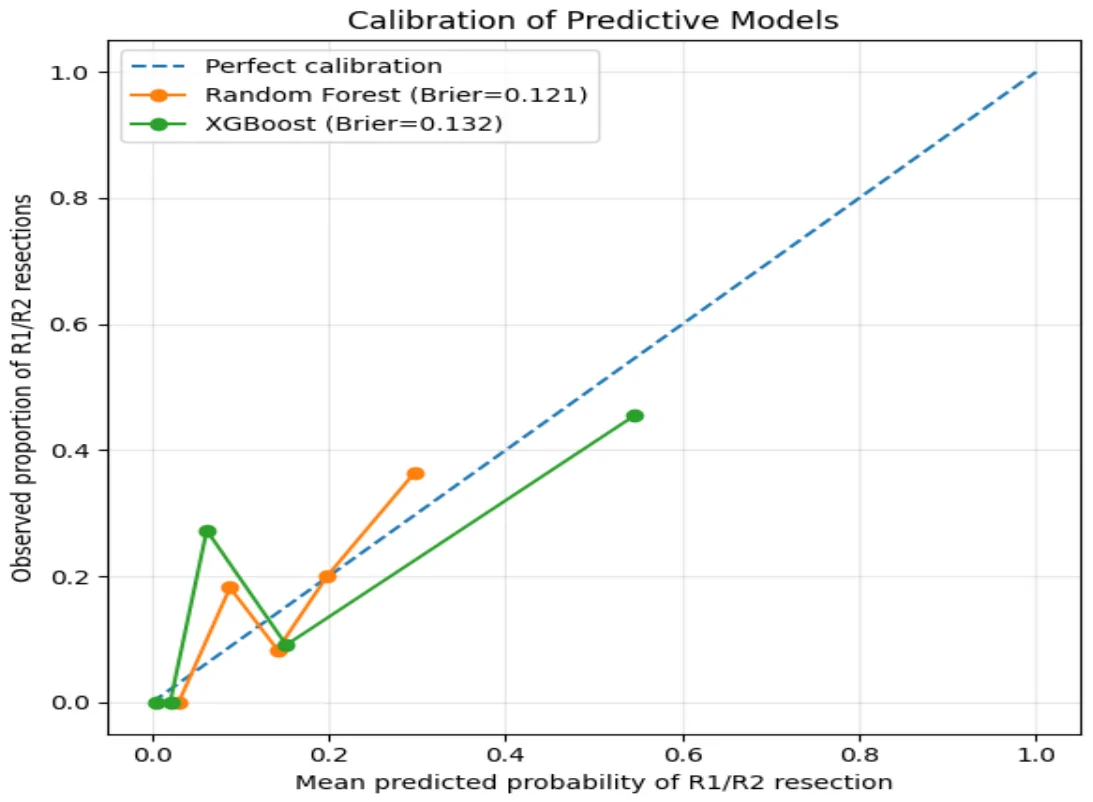
Calibration curves for the Random Forest (RF) and Extreme Gradient Boosting (XGB) models for prediction of R1/R2 resection. The dashed diagonal represents perfect agreement between predicted probabilities and observed event rates. Calibration was assessed using five quantile-based probability groups. Brier scores were 0.121 for RF and 0.132 for XGB.

Calibration analysis demonstrated Brier scores of 0.121 for RF and 0.132 for XGB, indicating slightly better overall probabilistic accuracy for RF. Calibration curves showed approximate agreement between predicted and observed probabilities across several probability ranges, although deviations from perfect calibration were observed for both models (Figure 6). These findings should be interpreted cautiously given the limited size of the held-out test set (n = 55) and the small number of R1/R2 events (n = 9). Given the limited number of R1/R2 events in the test cohort, these findings should be regarded as exploratory and reinforce the need for calibration assessment and external validation in substantially larger multicenter cohorts before clinical implementation.

Given the biological and therapeutic heterogeneity of the included malignancies, an additional subgroup analysis was performed to assess the association between tumor origin and resection-margin status. R0 resection was achieved in 69 of 90 patients with gynecologic malignancies (76.7%), 21 of 27 patients with colorectal malignancies (77.8%), and 92 of 103 patients with urologic malignancies (89.3%). The overall distribution of R0 versus R1/R2 resections differed significantly according to tumor origin (Fisher–Freeman–Halton exact *p* = 0.0426). These findings indicate that tumor origin contributes to the probability of achieving complete resection and therefore represents a relevant component of the predictive signal captured by the models.

## 4. Discussion

The survival outcomes observed in the present cohort are broadly consistent with the ranges reported in the contemporary pelvic exenteration literature. Kaplan–Meier analysis demonstrated a median overall survival of 40 months and a median progression-free survival of 18 months, findings that compare favorably with most large institutional and multicenter series published over the last two decades. Historical studies initially reported considerably poorer oncologic outcomes following pelvic exenteration, with 5-year overall survival rates frequently below 20–30%, particularly in cohorts including recurrent or highly advanced tumors [26,27,28]. However, progressive improvements in imaging modalities, perioperative care, reconstructive techniques, vascular surgery, and multimodal oncologic treatment have gradually transformed pelvic exenteration from a predominantly palliative procedure into a potentially curative strategy for carefully selected patients [10,25,29].

One of the most important findings of the present study is the significantly superior survival observed in patients with colorectal malignancies compared with gynecologic and urologic tumors. Patients with colorectal cancers achieved a median overall survival of 52 months, substantially exceeding the survival observed in the remaining oncologic subgroups. These findings are strongly supported by the available literature, where colorectal malignancies consistently demonstrate the most favorable long-term outcomes after pelvic exenteration. Shirouzu et al. reported 5-year survival rates reaching 71% in patients with primary locally advanced colorectal tumors undergoing curative pelvic exenteration [30]. Similarly, Vermaas et al. demonstrated markedly superior oncologic outcomes in primary colorectal tumors compared with recurrent disease [31], while Nielsen et al. confirmed improved long-term survival in patients with primary advanced rectal cancer treated within specialized centers [32].

The superior outcomes observed in colorectal malignancies likely reflect both improved resectability and the increasing availability of multimodal treatment strategies. Colorectal tumors frequently present with more favorable anatomical patterns of loco-regional extension, facilitating en bloc radical resection while preserving technically achievable dissection planes. Furthermore, colorectal malignancies increasingly benefit from multimodal conversion strategies, including neoadjuvant chemoradiotherapy, staged metastasectomy, cytoreductive approaches, and aggressive management of oligometastatic disease [33]. Contemporary reviews and PelvEx Collaborative publications similarly emphasize that high-volume specialized centers can achieve long-term survival rates exceeding 40–50% in selected colorectal patients undergoing exenterative surgery [25,34].

Another major finding of the present study is the pronounced survival difference between primary and recurrent disease. Patients undergoing pelvic exenteration for primary tumors achieved a median overall survival of 44 months, whereas recurrent disease was associated with a median survival of only 19 months. This observation closely mirrors the trends consistently reported throughout the literature. Ferenschild et al. reported 5-year survival rates of 66% for primary colorectal tumors compared with only 8% for recurrent disease [35]. Similarly, Vermaas et al. and Nielsen et al. demonstrated substantially poorer long-term outcomes in recurrent pelvic tumors compared with primary locally advanced disease [31,32]. Comparable findings have also been reported in gynecologic oncology, where recurrent cervical and endometrial cancers remain associated with inferior oncologic outcomes despite technically aggressive surgery. This is particularly relevant in cervical cancer, where contemporary management of locally advanced disease relies heavily on multimodal treatment, including concurrent chemoradiotherapy and image-guided adaptive brachytherapy [36].

The markedly poorer prognosis associated with recurrent disease is likely multifactorial. Previous surgery, radiotherapy, and systemic therapies produce extensive fibrosis, altered tissue planes, impaired vascularization, and inflammatory changes that substantially increase technical complexity and reduce the probability of achieving R0 resection. In addition, recurrent tumors frequently reflect biologically aggressive and treatment-resistant disease phenotypes. The strong association between primary tumors, higher rates of complete resection, and improved survival observed both in our cohort and in the available literature further reinforces the concept that careful patient selection and accurate preoperative assessment of resectability remain fundamental determinants of oncologic benefit following pelvic exenteration.

The subgroup analysis of total pelvic exenteration further illustrates the relationship between surgical extent and oncologic radicality. Patients undergoing total pelvic exenteration had a significantly lower R0 resection rate than those undergoing other exenterative procedures (62.2% vs. 88.0%), likely reflecting the greater local extent and anatomical complexity of the disease requiring complete pelvic clearance. Nevertheless, this difference did not translate into significantly inferior OS, while PFS was only modestly reduced, suggesting that extensive surgery may still provide meaningful oncologic benefit in appropriately selected patients despite the lower probability of achieving R0 resection.

Against this clinical background, interest has progressively shifted toward the development of predictive frameworks capable of improving preoperative estimation of resectability and supporting multidisciplinary therapeutic decision-making, particularly in procedures associated with major morbidity and functional consequences [25,34].

The RF model achieved the highest overall accuracy (83.6% versus 80% for XGB). However, class-specific analysis revealed that RF failed to identify cases with positive resection margins (R1/R2), with a recall of 0 for this category. RF therefore predominantly predicted R0 resections, explaining its apparently elevated accuracy while substantially limiting its clinical relevance. In contrast, XGB demonstrated a more balanced discriminatory capacity, achieving a recall of 33% for R1/R2 cases and a superior ROC-AUC (0.792 versus 0.767 for RF). Although sensitivity remained moderate, XGB was more effective in identifying patients at increased risk of incomplete resection.

Analysis of variable importance demonstrated distinct profiles between the two algorithms. RF predominantly prioritized preoperative biological parameters, which appear more closely related to general surgical risk than to the feasibility of radical oncologic resection. Conversely, although XGB also incorporated certain biological parameters, it placed greater emphasis on tumor type and extent, preoperative complications, comorbidities, and previous treatments. Poorly differentiated or recurrent tumors, metastatic disease, extra-pelvic extension, intraperitoneal fluid, gynecologic origin, previous pelvic radiotherapy or abdominal surgery, and complex pelvic fistulas were associated with a lower probability of R0 resection. Total pelvic exenteration and additional extra-pelvic organ resection were similarly associated with reduced R0 rates.

Interestingly, iliac vascular involvement and pelvic floor extension were not identified as negative predictors of R0 resection, potentially reflecting the technical resectability of these structures in experienced centers. In contrast, systemic dissemination and diffuse disease extension were more strongly associated with incomplete resection. These findings suggest that technically demanding but resectable anatomical involvement should not necessarily be regarded as equivalent to disease extension toward structures that preclude complete oncologic clearance. Accordingly, vascular or pelvic floor involvement alone should not automatically exclude a patient from consideration for pelvic exenteration when appropriate surgical expertise is available.

An important point of convergence between the two models was the identification of loco-regional lymphatic disease on preoperative imaging as a major predictor of R0 resection. The absence of radiologically visible lymphadenopathy is consistent with more localized disease and was associated with a higher probability of complete resection. Notably, both RF, which predominantly captured biological variables, and XGB, which placed greater emphasis on anatomical and oncologic factors, ranked this feature highly, suggesting a robust predictive signal across algorithmic approaches. Previous studies have similarly associated the absence of extensive nodal involvement with higher rates of radical resection and improved long-term survival [37,38,39]. Given the established relationship between R0 resection and survival, preoperative nodal status therefore warrants further investigation as a potential component of future patient-selection models.

The results of the present study suggest that tree-based predictive models exhibit different performance characteristics depending on the type of clinical signal captured. Random Forest demonstrated robustness but predominantly captured biological and metabolic status while tending to underrepresent the oncologic dimension of prediction. This tendency likely explains its apparently superior overall accuracy together with its complete lack of sensitivity for identifying R1/R2 cases. From a clinical perspective, such a model is insufficient because surgical decision-making fundamentally depends on the ability to anticipate incomplete resections.

XGB, through its regularization mechanisms and capacity to capture complex interactions, more faithfully incorporated clinico-anatomical predictive signals. Consequently, it appears to better reflect real-world surgical oncology practice, where loco-regional tumor extension and invasion of critical structures constitute the principal determinants of surgical radicality. Compared with RF, XGB demonstrated greater consistency with the available literature and with established oncologic principles, namely that anatomical extension and tumor dissemination determine the feasibility of radical surgery and therefore represent essential elements for identifying patients likely to achieve an optimal risk–benefit balance following pelvic exenteration.

Although its ability to recognize positive-margin cases remained imperfect, XGB provided a substantially more clinically relevant decision-support framework than RF, which failed entirely to identify this subgroup. Furthermore, the profile of important variables identified by XGB may provide the conceptual basis for future clinical selection algorithms integrating imaging findings, anatomical extension, and patient comorbidity into a structured preoperative risk-stratification framework.

Beyond oncologic radicality, patient selection for pelvic exenteration must also consider the balance between the anticipated therapeutic benefit and the substantial morbidity associated with extensive multivisceral surgery. In selected patients, the potential benefit of aggressive surgery may extend beyond curative intent; Tatar et al. reported meaningful symptomatic improvement following palliative pelvic exenteration combined with iliofemoral vascular reconstruction in a patient with advanced recurrent cervical cancer [40]. More broadly, experience from aggressive cytoreductive surgery for ovarian cancer suggests that the extent of surgery alone does not necessarily translate into prohibitive postoperative morbidity when complex procedures are performed in appropriately selected patients [41]. These observations reinforce the importance of individualized multidisciplinary assessment, in which technical resectability, expected oncologic or symptomatic benefit, patient condition, and anticipated surgical morbidity are considered together.

Based on the predictive elements identified by the XGB model, Table 3 proposes the following patient-selection and therapeutic decision-making algorithm:

## 5. Conclusions

The absence of radiologically visible regional lymphadenopathy emerged as an important predictor of R0 resection, reinforcing the central role of careful assessment of loco-regional disease extent in patient selection for pelvic exenteration. Conversely, vascular or pelvic floor involvement did not emerge as absolute limiting factors, suggesting that technically resectable anatomical extension should not necessarily preclude exenteration when appropriate surgical expertise is available. Ultimately, the oncologic value of pelvic exenteration remains strongly dependent on a realistic probability of achieving R0 resection, while alternative multimodal or palliative strategies should be considered when complete resection is unlikely.

The proposed classification into “R0-friendly”, “conversion”, and “low up-front probability of R0” categories should be regarded as an exploratory framework rather than a validated clinical decision tool. Although XGB identified clinically plausible patterns associated with resectability, its predictive performance, particularly for R1/R2 cases, remains limited. Validation in substantially larger multicenter cohorts is therefore required before clinical implementation. Future integration of clinical, anatomical, and advanced imaging data may enable the development of more robust models for individualized patient selection.

## Figures and Tables

**Table 1:** Univariable Analysis of Factors Influencing Overall Survival (OS) and Progression-Free Survival (PFS).

Variable	OS HR (95% CI)		PFS HR (95% CI)	
	Statistic	*p*-Value	Statistic	*p*-Value
Positive resection margins (R1/R2 vs. R0)	1.51 (1.04–2.19)	0.030	5.64 (3.68–8.64)	<0.0001
Emergency surgery (yes vs. no)	1.05 (0.59–1.90)	0.862	0.71 (0.31–1.63)	0.424
Extra-pelvic organ resection (yes vs. no)	1.06 (0.73–1.55)	0.754	1.93 (1.26–2.96)	0.0027
LAD (yes vs. no)	1.40 (0.96–2.03)	0.079	1.29 (0.83–2.00)	0.253
TNM stage (per category increase)	1.17 (0.94–1.45)	0.151	1.48 (1.12–1.95)	0.0062
Tumor differentiation grade (per grade increase)	1.02 (0.78–1.33)	0.897	1.03 (0.75–1.43)	0.841
Vascular involvement (yes vs. no)	1.21 (0.82–1.80)	0.336	1.65 (1.07–2.54)	0.0247
LVI (present vs. absent)	0.97 (0.71–1.32)	0.842	0.63 (0.42–0.93)	0.0213
PNI (present vs. absent)	1.00 (0.73–1.37)	0.996	0.57 (0.38–0.85)	0.0062
Lymph+ (present vs. absent)	1.03 (0.76–1.40)	0.859	0.49 (0.32–0.74)	0.00065

Abbreviations: LVI—lymphovascular invasion identified on the resection specimen; PNI—perineural invasion identified on the resection specimen; Lymph+—lymphocytic infiltrate identified on the resection specimen; LAD—regional lymphadenectomy.

**Table 2:** Comparative Summary of Predictive Model Performance Metrics.

Metric	Random Forest	Extreme Gradient Boosting
Overall accuracy	0.836	0.800
ROC-AUC	0.767	0.792
Precision (R0)	0.836	0.872
Sensitivity (R0)—recall	1.000	0.891
Specificity (R0)	0.000	0.333
F1-score (R0)	0.911	0.882
Precision (R1/R2)	0.000	0.375
Sensitivity (R1/R2)—recall	0.000	0.333
Specificity (R1/R2)	1.000	0.891
F1-score (R1/R2)	0.000	0.353
Balanced accuracy	0.500	0.612

**Table 3:** Proposed Patient-Selection Guide According to the Probability of Achieving R0 Resection.

Category	Clinical Profile	Recommended Management
**“R0-friendly” cases**	Absence of radiologically evident loco-regional lymphadenopathy No extra-pelvic extension Primary tumors with well or moderately differentiated histology	Standard surgical approach Profile associated with a higher probability of R0 resection (R0)
**“Conversion” cases**	Partially reversible adverse factors, including recurrent or poorly differentiated tumors (G3) Limited peritoneal dissemination Post-radiotherapy/post-surgical sequelae Absence of diffuse invasion Absence of bulky nodal disease	Neoadjuvant therapy (chemoradiotherapy), repeat imaging reassessment, and extended multidisciplinary tumor board discussion R0 resection may become achievable following conversion treatment
**Cases with low probability of R0 resection**	Evident regional lymphadenopathy Ascites Diffuse extra-pelvic extension Systemic metastases Absence of response to neoadjuvant therapy	Complete re-staging Palliative systemic treatment Consideration of conversion-treatment protocols in selected cases after discussion in tumor-board Avoidance of highly mutilating pelvic exenteration without realistic probability of achieving R0 resection

## Data Availability

The datasets presented in this article are not publicly available due to institutional and national legislative restrictions regarding privacy and data management.

## Abbreviations

The following abbreviations are used in this manuscript:

AI	Artificial Intelligence
APTT	Activated Partial Thromboplastin Time
AUC	Area Under the Curve
CI	Confidence Interval
CNAS	Romanian National Health Insurance House
CT	Computed Tomography
HR	Hazard Ratio
INR	International Normalized Ratio
LAD	Lymphadenectomy
LVI	Lymphovascular Invasion
ML	Machine Learning
MRI	Magnetic Resonance Imaging
OS	Overall Survival
PFS	Progression-Free Survival
PNI	Perineural Invasion
PT	Prothrombin Time
R0	Microscopically Negative Resection Margin
R1	Microscopically Positive Resection Margin
R2	Macroscopically Positive Resection Margin
RF	Random Forest
ROC-AUC	Area Under the Receiver Operating Characteristic Curve
TNM	Tumor–Node–Metastasis Classification
XGB	Extreme Gradient Boosting
